# Nrf2 regulates cell motility through RhoA–ROCK1 signalling in non-small-cell lung cancer cells

**DOI:** 10.1038/s41598-021-81021-0

**Published:** 2021-01-13

**Authors:** Eunsun Ko, Dasom Kim, Dong Wha Min, Seung-Hae Kwon, Ji-Yun Lee

**Affiliations:** 1 Department of Pathology, Korea University College of Medicine, 73, Goryeodae-ro, Seongbuk-gu, Seoul, 02841 South Korea; 2 Department of Biomedical Science, Korea University College of Medicine, Seoul, 02841 South Korea; 3 Seoul Center, Korea Basic Science Institute, Seoul, 02841 South Korea

**Keywords:** Cancer, Lung cancer, Non-small-cell lung cancer, RHO signalling, Cell signalling, RHO signalling, Cell migration, Cell invasion

## Abstract

Nuclear factor-erythroid 2-related factor 2 (Nrf2) is a key transcriptional regulator of several antioxidant and anti-inflammatory enzymes. It binds to its endogenous inhibitor Kelch-like ECH-associated protein 1 (Keap1) in the cytoplasm under normal conditions. Various endogenous or environmental oxidative stresses can disrupt the Nrf2/Keap1 complex, allowing Nrf2 to translocate into the nucleus, where it induces the transcription of various cytoprotective enzymes by binding to antioxidant responsive elements. These enzymes have been reported to play a role in regulating tumour growth, angiogenesis, and chemoprevention. Invasion and migration are the most harmful aspects of cancer; they directly impacts the patients’ survival. Although the roles of Keap1/Nrf2 and their downstream genes in various cancers have been widely documented, their role in regulating cell motility still remains unclear, particularly in cancer cells. We observed that Nrf2 suppression following treatment with brusatol in non-small-cell lung cancer (NSCLC) cells with either exogenously introduced Keap1 or siNrf2 resulted in the inhibition of cell migration and invasion, with shrinking cell morphology due to decreased focal adhesions via inhibition of the RhoA–ROCK1 pathway. Nrf2 overexpression showed opposite results. Thus, the Nrf2/Keap1 pathway may affect cell motility by dysregulating the RhoA–ROCK1 signalling pathway in NSCLC.

## Introduction

Carcinomas, which originate from epithelial cells, constitute more than 90% of all malignant human cancers; in carcinoma patients, lymph node and peritoneal metastasis is a major cause of tumour recurrence and cancer-related death^1^. Cell motility is necessary for the sequential multi-step processes involved in cancer metastasis, such as invasion, intravasation, and extravasation. The ability of cancer cells to invade into the surrounding tissues is one of the major hallmarks of cancer, which requires increased cell motility driven by remodelling of the cytoskeletal system and the contact of the cells with the extracellular matrix. This acquired migratory and invasive ability of cancer cells during metastasis is similar to the epithelial to mesenchymal transition (EMT) that occurs during embryonic development, tissue remodelling, and wound healing^2–4^. Several mechanisms associated with EMT have been studied. One of the most well-studied mechanisms of EMT is the signalling pathway driven by growth factors associated with receptor tyrosine kinases (RTK) and other signalling proteins such as TFG-β, and Wnt/β-catenin-activated downstream transcriptional repressors of E-cadherin^5,6^. The other effectors of cell motility are modulators of other adhesion systems and activators of actin cytoskeleton remodelling, such as Rho-family GTPases^7,8^.

Nuclear factor-erythroid 2-related factor 2 (Nrf2) is an important transcriptional regulator of many antioxidant and anti-inflammatory enzymes. Nrf2 binds to its endogenous inhibitor, Kelch-like ECH-associated protein 1 (Keap1) in the cytoplasm under normal conditions. The Nrf2–Keap1 complex can be disrupted by various endogenous or environmental oxidative stresses, which leads to accumulation and transactivation of Nrf2. Recently, increasing number of studies have shown and continue to show that persistent Nrf2 activation due to dysregulation of the Nrf2–Keap1 pathway in various cancer cells induces cell proliferation/growth by reprogramming metabolic processes. This is associated with poor prognosis due to acquired resistance to chemotherapy^9,10^. However, limited studies have shown the role of Nrf2/Keap1 in the regulation of cell motility and EMT in cancer cells and hence, it remains unclear^11–15^.

Our previous study showed that overexpression of Nrf2 due to Keap1 mutation increased cell invasion and metastatic ability of EGFR tyrosine kinase inhibitor (TKI)-resistant lung cancer cells both in vitro and in vivo. We also observed suppression of RhoA, ROCK1, Snail, and β-catenin, which are all well-known markers associated with cell motility/movement and EMT^16^. Therefore, in this study, we further investigated the mechanism underlying the induction of cell motility by the Nrf2–Keap1 pathway in lung cancer cells.

## Results

### Nrf2 regulates the motility of NSCLC cells

**Figure 1:**
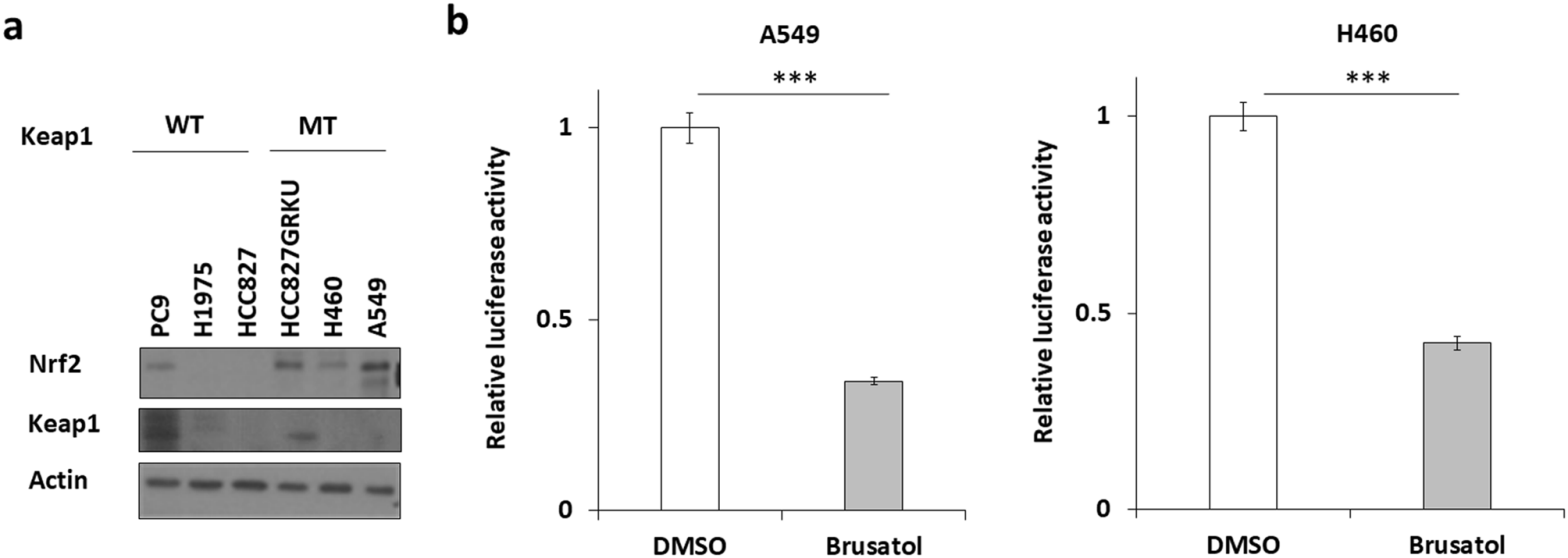
Expression levels of Nrf2 and Keap1 in NSCLC cells and inhibitory effect of brusatol on Nrf2 expression. (**a**) Nrf2 and Keap1 expression levels in different types of NSCLC cells were evaluated using Western blotting. (**b**) Inhibition of Nrf1 transcriptional activity seen after evaluation of ARE using dual luciferase assay after treatment with brusatol in H460 (10 nM) and A549 (30 nM) cells for 24 h.Expression levels of Nrf2 and Keap1 in NSCLC cells detected by western blotting.

**Figure 2:**
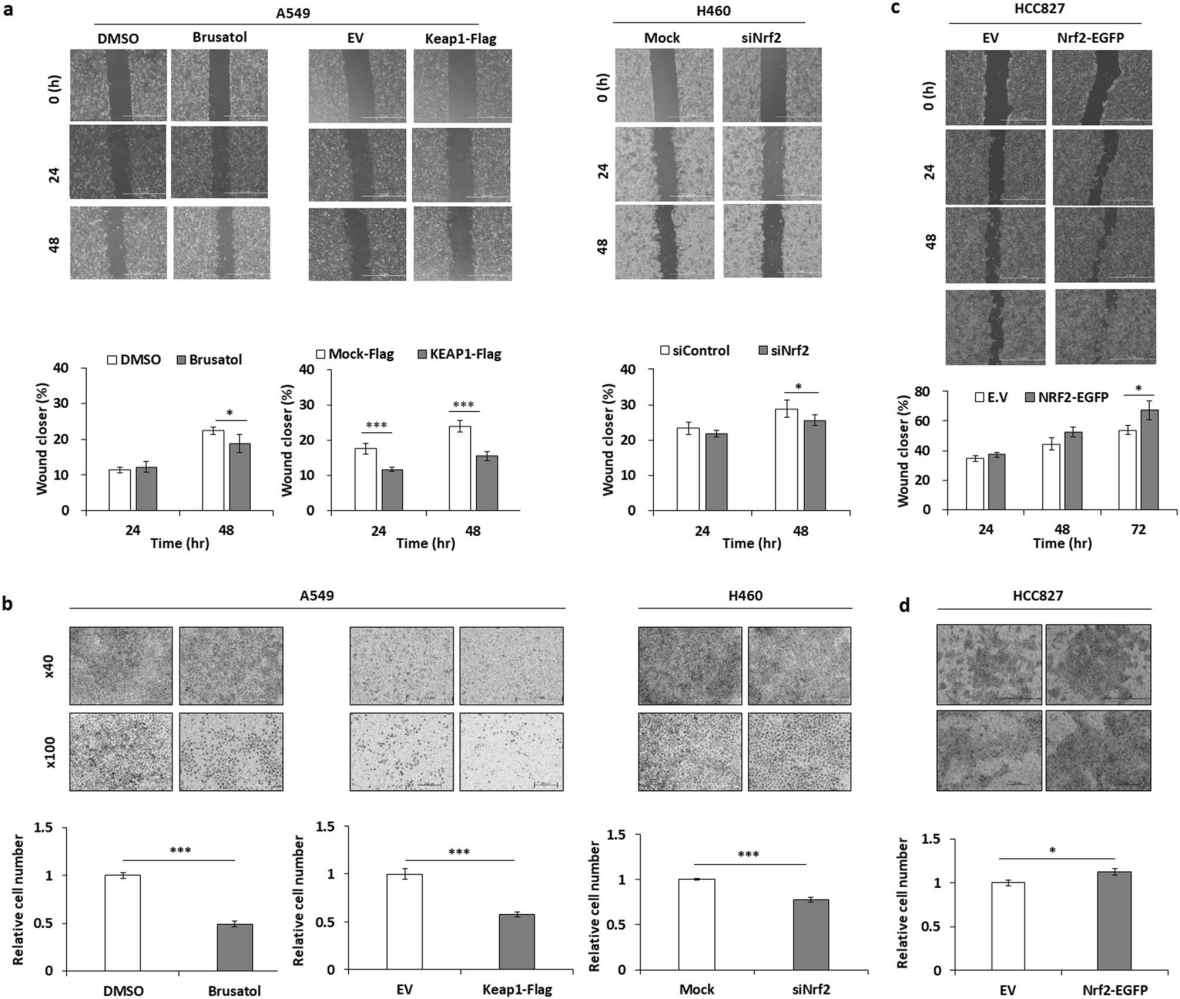
Nrf2 regulates cell motility. Nrf2 expressed was suppressed in A549 or H460 cells following treatment with brusatol (10 nM) or transfection of Keap1-Flag or siNrf2. (**a**,**c**) Horizontal migration of these cells was compared with that of the control cells using wound healing assay for the indicated time points. (**b**,**d**) Nrf2 was overexpressed by transfecting Nrf2-EGFP into the HCC827 cells. Vertical migration was detected by transwell assay after brusatol treatment or transfection for 24 h. The degree of migration was quantified by calculating the area of migrated cells using the image processing software, Image J (Ver. 1/52n, NIH, Bethesda, MD, USA). Scale bar: 200 µm *p < 0.05, **p < 0.01, ***p < 0.001 compared with control.

To investigate whether Nrf2 affects cell motility, migration and invasion assays were performed. Wild-type Keap1-Flag or siNrf2 was introduced into A549 and H460 cells, which harbour Keap1 mutations, and hence, have a higher expression of Nrf2 compared to the cells without Keap1 mutations (Fig. 1a). These cells were then treated with brusatol, an Nrf2 inhibitor, and consequently, inhibition of cell migration and invasion was observed (Fig. 2a,Bb). The effect of brusatol was confirmed using dual luciferase assay (Fig. 1b). In addition, we observed that overexpression of Nrf2 by exogenously introducing Nrf2-EGFP into the HCC827 cells increased the migration and invasion ability of these cells (Fig. 2c,d). These results indicated that Nrf2 plays a role in regulating cell motility.

### RhoA–ROCK1 pathway is associated with Nrf2-induced increase in NSCLC cell motility

**Figure 3:**
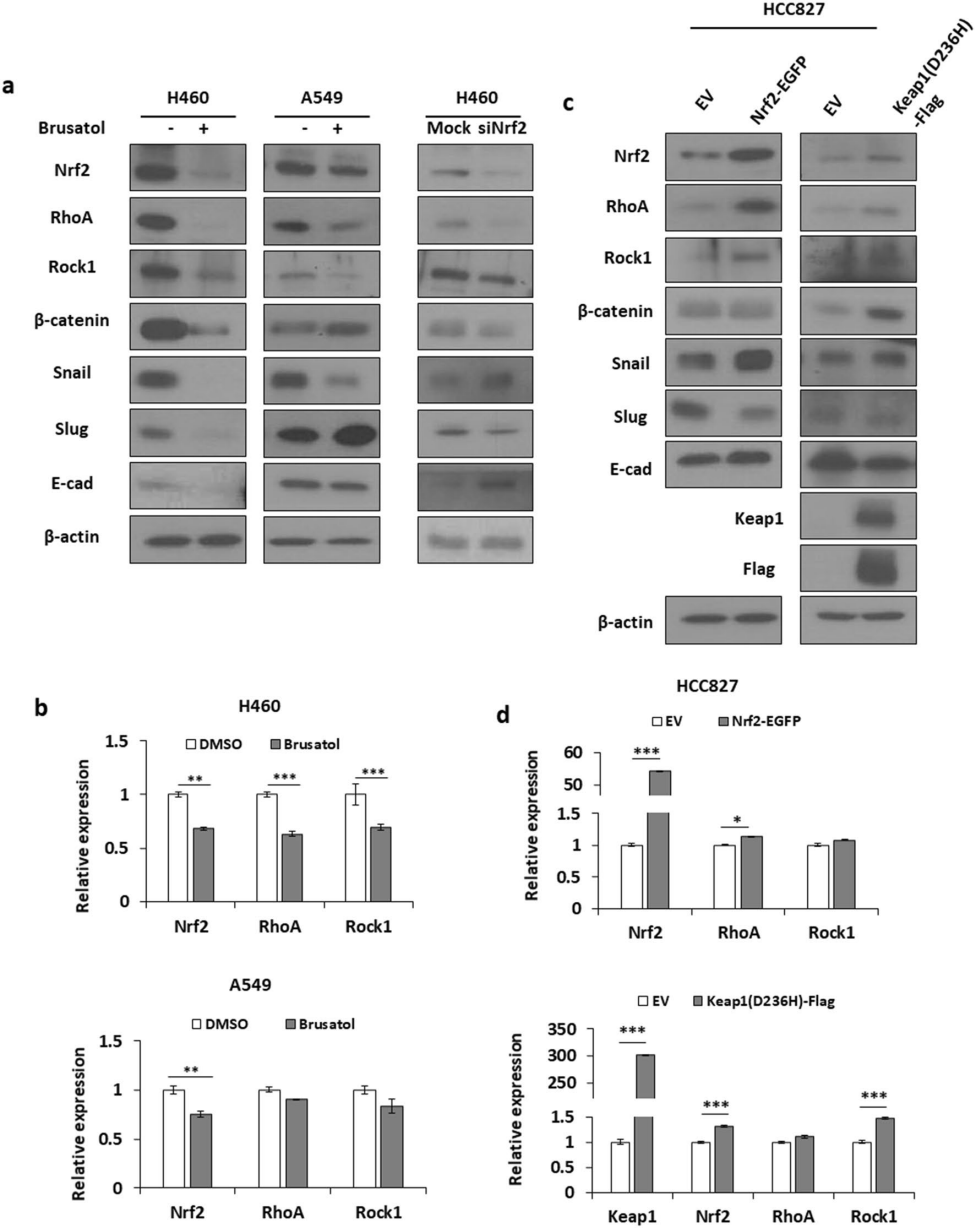
Molecules from the RhoA-ROCK1 pathway, and not the EMT-related molecules, are associated with Nrf2-induced cell motility. The expression levels of Nrf2, RhoA, Rock1, and/or EMT markers were detected using (**a**,**c**) western blotting or (**b**,**d**) qRT-PCR. (**a**,**b**) Nrf2 was suppressed by treating the A549 or H460 cells with brusatol (10 nM) or transfection of Keap1-Flag or siNrf2. (**c**,**d**) Nrf2 was overexpressed by transfecting HCC827 cells with Nrf2-EGFP or Keap1 mutant (D236H)-Flag for 24 h.*p < 0.05, **p < 0.01, ***p < 0.001 compared with control.

To investigate the molecular pathway utilised by Nrf2 in the regulation of cell motility, the levels of EMT-related proteins, such as Snail, Slug, and E-cadherin, and cytoskeleton-related RhoA–ROCK1 pathway molecules were examined by western blotting and/or qRT-PCR. Inhibition of Nrf2 after brusatol treatment resulted in a consistent decrease in the expression levels of RhoA and ROCK1 proteins in A549 and H460 cells (Fig. 3a,b). The opposite effect was seen in the HCC827 cells when Nrf2 was overexpressed by exogenously introducing Nrf2–EGFP and the Keap1 mutant, Keap1 (D236H)-Flag (Fig. 3c,d). However, the mRNA level of expression of RhoA and ROCK1 was not consistent with protein level of expression, which suggests the possibility of post-translational modification of RhoA and ROCK1. We also observed that the expression levels of EMT-related molecules after suppression or overexpression of Nrf2 varied depending on the cell lines irrespective of the presence of Nrf2 (Fig. 3a,b). These results indicated that Nrf2 regulates cell motility through the RhoA–ROCK1 pathway, and not through the EMT pathway.

### Stability and/or activity of RhoA is associated with Nrf2-induced increase in NSCLC cell motility

**Figure 4:**
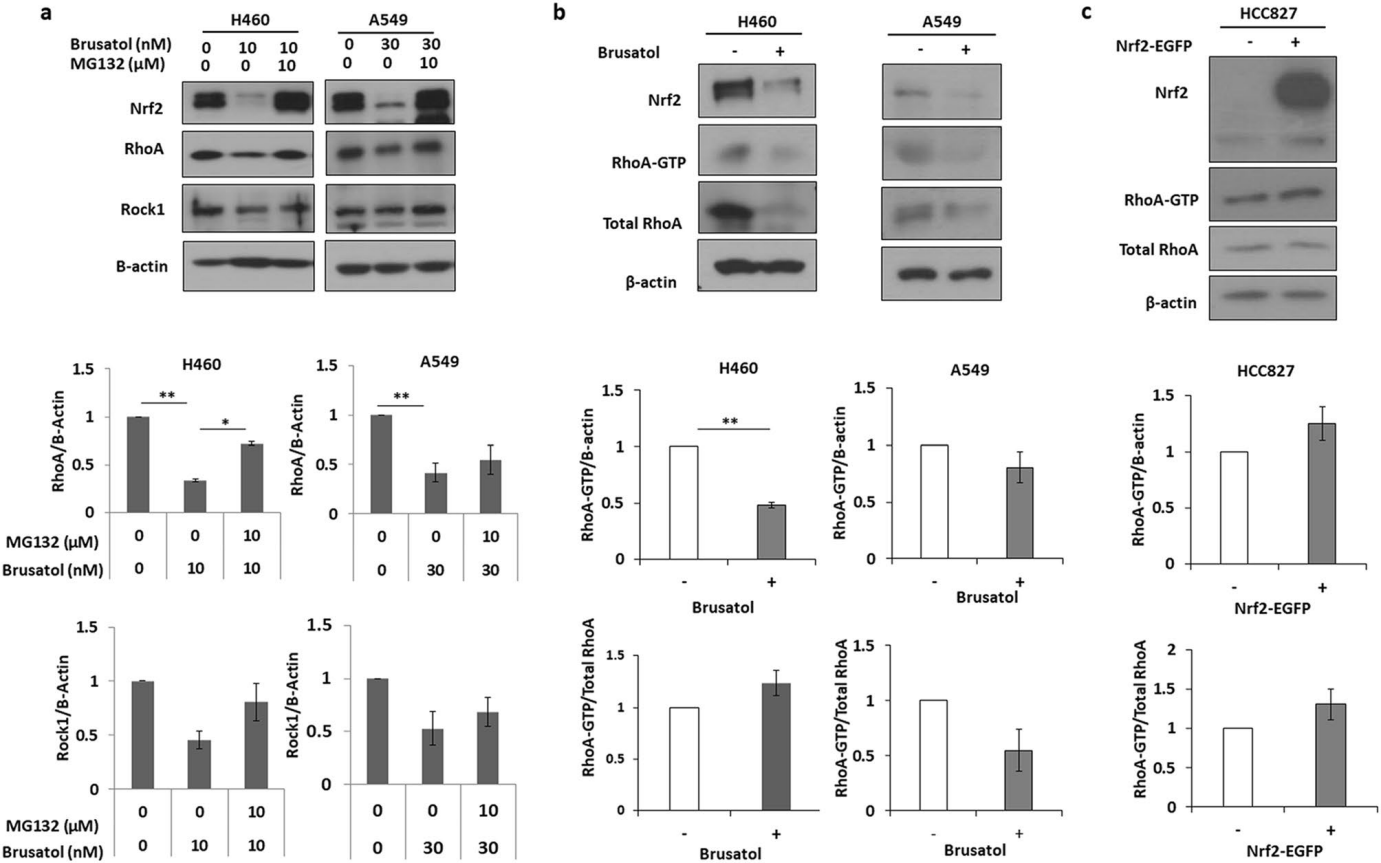
Nrf2 increases the stability or activity of RhoA. (**a**) Cells were treated with MG132 with or without brusatol for 3 h and then harvested for western blotting. (**b**,**c**) Activation of RhoA was measured using RhoA Pull-down activation assay. RhoA-GTP was detected by western blotting. A549 and H460 cells were treated with brusatol (15 nM and 10 nM) for 24 h. HCC827 was transfected by exogenously introduced Nrf2-EGFP for 24 h. The densitometry quantification of the western blot determined using Image J software (Ver. 1/52n, NIH).

Since it has been shown that Nrf2 controls the RhoA–ROCK1 pathway, the mechanism how Nrf2 affects the RohA–ROCK1 signal axis beyond changes in total protein was investigated. H460 and A549 cells treated with MG132 to inhibit proteasomal degradation. The result showed RhoA and ROCK1 expression was recovered by treatment of MG132 with brusatol in H460 indicating Nrf2 controls RhoA–ROCK1 pathway through proteosomal degradation, but not in A549 cells (Fig. 4a). Besides, the effect of Nrf2 on the activity of RhoA was evaluated using GST pulldown assay. Brusatol-mediated inhibition of Nrf2 induced down-regulation of the active RhoA-GTP form in the A549, but not in H460 cells (Fig. 4b), and overexpression of Nrf2 by exogenously introducing Nrf2 resulted in a rise in the RhoA-GTP levels in HCC827 cells (Fig. 4c). These findings suggest that Nrf2 can regulate the RhoA–ROCK1 pathway through increased level of RhoA-GTP induced by the stability of RhoA and/or activity of RhoA-GTP in NSCLC cells, which is associated with protein level of RhoA.

### Nrf2 regulates the formation of actin stress fibres

**Figure 5:**
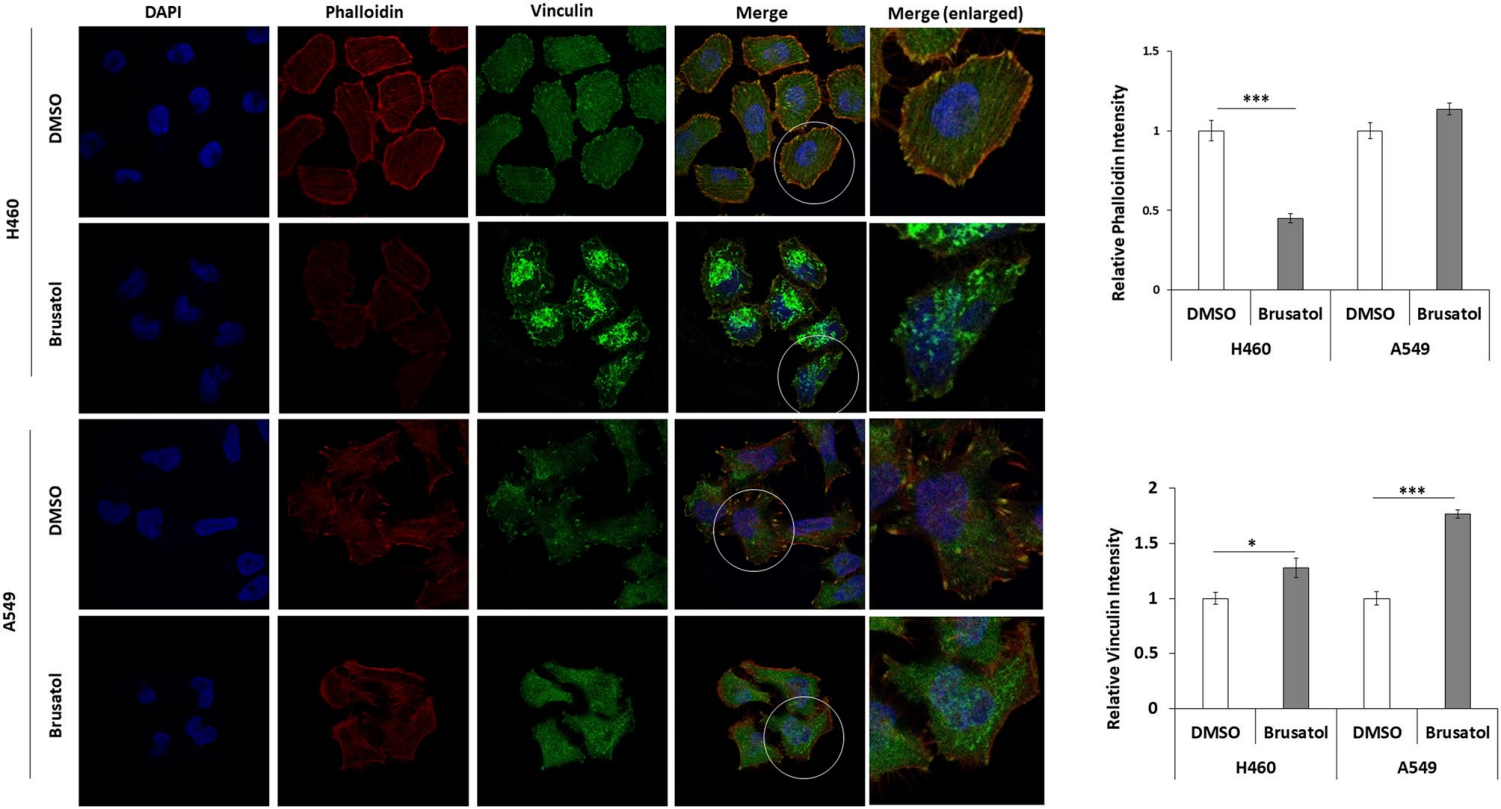
Nrf2 is associated with the formation of F-actin stress fibres with focal adhesion. Representative images of cells stained with vinculin (green) with rhodamine phalloidin (red) after brusatol treatment (30 nM and 10 nM for A549 and H460 cells, respectively). White circled cell enlarged. Nuclei were counterstained with DAPI (blue). Images were obtained with a confocal laser scanning microscope (LSM 780, ZEISS) and visualised using the 100× oil immersion objective (EC Plan-Neofluar 100×/1.3 oil). Intensity of fluorescence from the captured image of cells was quantified using ImageJ software (Ver. 1/52n, NIH).

To demonstrate the effect of Nrf2 on cell motility, we observed the morphology and distribution of the F-actin cytoskeleton along with focal adhesions (Fig. 5). F-actin was stained with rhodamine phalloidin (red) and the focal adhesions were detected by vinculin staining after the inhibition of Nrf2 via brusatol treatment in the H460 and A549 cells. Results showed the presence of diffused short rod-like F-actin (red) with reduced focal adhesion (yellow) with increased expression of vinculin, throughout the cytoplasm and the appearance of shrinking cell morphology, while the control cells exhibited noticeably stretched stress fibre formations. This indicated the down-regulation of the formation of stress fibres, as well as a significant decrease in the formation of focal adhesions.

## Discussion

Cell migration plays a crucial role in many types of human diseases including cancer, where dysregulation of cell migration can lead to metastasis, resulting in the poor prognosis of cancer patients. Investigation of genes or pathways that regulate cell migration and metastasis will facilitate the understanding of this process and the development of novel diagnostic and treatment strategies for cancer. Nrf2 is a transcription factor that has been known to be a master regulator of the cytoprotective response towards oxidative and electrophilic stresses. Under basal conditions, Nrf2 is localised in the cytoplasm by a cluster of proteins that degrade it quickly. Under oxidative stress, instead of being degraded, Nrf2 is translocated to the nucleus, where it binds to DNA promoters and initiates the transcription of several anti-oxidant genes and their proteins in healthy normal cells^17,18^. In contrast to the cytoprotective role of Nrf2 in normal cells, accumulating evidence suggests that persistently high activation of Nrf2 in various type of cancers, including lung cancer, is related with progression, metastasis, and resistance against chemotherapy and radiotherapy, which leads to poor prognosis^16,19,20^. The Keap1–Nrf2 pathway is therefore, considered to be a novel oncogenic signalling pathway and an attractive target for developing anti-cancer therapeutics. However, although the role of Keap–Nrf2 is well studied, studies on the role of Nrf2 in cell motility, EMT, and the underlying signalling pathways in cancer cells are scarce^11–15^.

In this study, we further investigated how Nrf2 regulates motility in NSCLC cells based on our previous findings, which showed that high activation of Nrf2 due to Keap1 mutation promotes migration ability both in vitro and in vivo in EGFR-TKI resistant lung cancer cells. Well-known EMT makers such as Snail, Slug, and E-cadherin, were examined in Nrf2-overexpressing or -knockdown NSCLC cells. Results showed that the expression of these molecules was not consistent, which might explain why previously published reports have shown controversial and cell-type specific results^21–24^. Other molecules associated with cytoskeletal remodelling such as RhoA and ROCK1 were also examined and we observed that overexpression of Nrf2 up-regulated, and suppression of Nrf2 down-regulated the RhoA–ROCK1 pathway. The RhoA/ROCK1 pathway plays an important role in cell movement and morphogenesis, which is associated with invasion and metastasis, as cellular cytoskeletal regulation and reprogramming is one of its main functions^8,25^. RhoA, which is a member of the Rho family of GTPases, facilitates the polymerisation of actin (F-actin formation) to form stress fibres, which are antiparallel actin filaments that are crosslinked by myosin, as well as the activation of myosin to trigger cell contractility. Binding of GTP-bound active RhoA activates ROCK1, which induces actin-myosin contraction by stimulating the phosphorylation of the myosin light chain directly. Additionally, the contractile force of the actin-myosin network is used to pull on the extracellular matrix (ECM) at the focal adhesion sites, while ECM stiffness can facilitate the formation of focal adhesions to allow cell movement. Increased level of RhoA-GTP was verified by the increase in the levels of RhoA stability and/or activity of RhoA-GTP following Nrf2 overexpression and decrease in RhoA-GTP levels after Nrf2 suppression in NSCLC cells. Moreover, immunostaining of F-actin with vinculin after Nrf2 inhibition by brusatol resulted in the increased expression of vinculin, which is known to inhibit cellular motility^26^ and formation of diffused short rod-like F-actin with reduced focal adhesions throughout the cytoplasm, along with the appearance of shrunken cell morphology, while the control cells exhibited noticeable formation of stretched stress fibres, indicating that Nrf2 activated the formation of actin stress fibres, leading to increased cell motility^27^. Similar results were reported in breast and gastric cancer cells, where it was seen that Nrf2/Keap1 promoted cell migration by upregulating the RhoA/ROCK1 pathway^11,28^. In lung adenocarcinoma cells, it has been reported that Keap1–Nrf2 interactions suppressed cell motility by targeting S100P^15^. In addition, this report suggested the possibility of an association of RhoA in the Nrf2/Keap1 pathway with the motility of lung cancer cells, as significant morphological and cytoskeletal changes were noticed due to the manipulation of Keap1 and suppression of RhoA activity by Keap1 overexpression in Cl1 lung cancer cells^15^. These results are consistent with ours. Taken together, we conclude that the Nrf2–Keap1 complex regulates cancer cell motility through the RhoA–ROCK1 signalling pathway, and not via EMT related molecules, in lung cancer cells.

## Methods

### Cell culture and reagents

The human NSCLC cells cell lines HCC827, H460, and A549 were purchased from American Type Culture Collection (ATCC, USA). These cells were cultured in RPMI1640 (Welgene, Daegu, South Korea) supplemented with 10% foetal bovine serum (FBS) (Welgene, Daegu, South Korea) and 1% penicillin/streptomycin at 37 ℃ in a humidified atmosphere containing 5% CO_2_. Brusatol (Carbosynth, UK) was purchased and dissolved in dimethyl sulfoxide (DMSO) (D2650, Sigma Aldrich, USA). MG132 was purchased from CalBiochem (SanDiego, CA, USA). Nrf2-EGFP and Keap1-Flag vectors were purchased from Addgene (http://www.addgene.org). The oligo ribonucleotide sequences of human Nrf2 siRNA (siNrf2) were as follows: 5′-UCUGACUCCGGCAUUUCACUTT-3′ (sense) and 5′-AGUGAAAUGCCGGAGUCAGATT-3′ (antisense) (Shanghai GenePharma Co. Ltd., China).

### Cell migration assay

Cell migration was measured using wound healing assay. Cells were seeded into 6-well plates and cultured to a confluent monolayer for 24 h. The cell monolayer was scratched using a 200-µl sterile pipette tip and washed twice with phosphate buffered saline (PBS) to remove the detached cells. Following hours of culture in RMPI-1640 supplemented with 1% serum to minimize cell proliferation during the period of assay^29^. Initial images of four independent areas after each scratch were acquired at time zero. Each scratch was examined, the images of the same location were captured, and the healed area was measured after the indicated time. The degree of migration was quantified by calculating the area of migrated cells using the image processing software, Image J (Ver. 1/52n, NIH, Bethesda, MD, USA).

### Transwell invasion assay

The invasiveness of tumour cells was evaluated using an invasion assay with Transwell devices (CT-3422, 8 μm pore size, 6.5 mm diameter, Corning Life Science, USA) coated with Matrigel (BD, 356230, 100 μg/ml, 15 µl/well). Cells (8 × 10^4^) were placed in the upper chamber of the Transwell device in serum-free media. Media containing 10% FBS was added as a chemoattractant in the lower chamber of each well. Non-invading cells were removed using cotton swabs. The remaining cells were fixed and stained by Hemacolor rapid staining solutions (Merck, Kenilworth, NJ, USA) for 3 min. The numbers of invasive cells were counted from five representative fields on the membrane.

### RNA isolation and quantitative real time polymerase chain reaction (qRT-PCR)

Total RNA was isolated from the cells using TRIzol reagent (Invitrogen, Carlsbad, CA, USA). cDNA was synthesised from the total RNA using the reverse transcription kit (LaboPass, Cosmo Genetech, Seoul, South Korea) according to the manufacturer’s instructions. qRT-PCR was carried out using gene specific primers with SYBR green Q master mix (Labopass, Cosmo Genetech, Seoul, South Korea) on an ABI7500 real-time PCR system (Applied Biosystems, Warrington, UK). The oligonucleotide primers that were used for the qRT-PCR are described in Table 1. The C_t_ values of the target genes were normalised to an endogenous reference gene (GAPDH). Each gene was analysed in duplicate and the analysis was repeated via three independent experiments.

**Table 1:** Oligonucleotides for qRT-PCR.

Gene	Forward	Reverse
RhoA	5′-GCAGATATCGAGGTGGATGG-3′	5′-CTATCAGGGCTGTCGATGG-3′
Rock1	5′-TGAGGTTAGGGCGAAATGGT-3′	5′-AATCGGGTACAACTGGTGCT-3′
Nrf2	5′-TTGAGCAAGTTTGGGAGGAG-3′	5′-AGTTTGGCTTCTGGACTTGG-3′
Keap1	5′-TGTTACAACCCCATGACCAA-3′	5′-CGCTCTGGCTCATACCTCTC-3′
GAPDH	5′-ACCCACTCCTCCACCTTTGA-3′	5′-CTGTTGCTGTAGCCAAATTCGT-3′

### Western blot analysis

Proteins from the cell lysates were separated using SDS-PAGE and transferred onto Immobilon-P PVDF membranes (IPVH00010, Millipore, USA). These membranes were subsequently probed with the appropriate primary antibodies and incubated with the corresponding goat anti-mouse Ig G (7076) or goat anti-rabbit Ig G (7074) secondary antibodies conjugated with horseradish peroxidase (HRP). Chemiluminescence was detected using the enhanced chemiluminescence (ECL) system (Translab, South Korea) according to the manufacturer’s instructions. Secondary antibodies were purchased from Cell Signaling technology, Inc. The primary antibodies against Snail (3879), Slug (9585), E-cadherin (3195), β-catenin (9582), and Keap1 (7705) were purchased from Cell Signaling technology. Antibodies against ROCK1 (sc-5560), RhoA (sc-179), and β-actin (sc-47778) were purchased from Santa Cruz Biotechnology. Antibodies against Nrf2 (ab62352) were purchased from Abcam. Anti-Flag antibodies (F1804) were purchased from Sigma Aldrich. The result of gels images were cropped and full-length gels and blots are included in the Supplementary Figures.

### GST-Rhotekin-RBD pull-down assay

The activation of RhoA was measured using the RhoA Pull-down Activation Assay Biochem kit (BK036, Cytosekeleton, USA) according to the manufacturer’s protocol. Cells were harvested and washed with PBS and then lysed in ice-cold cell lysis buffer containing 1× protease Inhibitor Cocktail. The lysates were centrifuged at 10,000 rpm for 2 min at 4 ℃. The supernatant (500 µg) was incubated with GST-Rhotekin-RBD to detect RhoA-GTP. The beads were washed thrice with washing buffer. The bound proteins were eluted with 2× Laemmli sample buffer and loaded onto an SDS-PAGE gel. The primary anti-RhoA antibody was provided in the kit.

### Immunofluorescence

Cells (2 × 10^2^ cells/well) were plated in a 6-well-plate containing coverslips in 2 ml of culture media and cultured overnight. The cells were washed twice with PBS and then fixed with 4% formaldehyde in PBS at room temperature (RT) for 30 min. The fixed cells were washed thrice with PBS for 5 min. After washing, the cells were permeabilised with 0.1% TritonX-100 in PBS for 15 min, followed by three washes with PBS. The fixed cells were blocked with 3% (w/v) BSA, stained with an anti-Vinculin antibody (Abcam, CA, Cat No. ab129002) and rhodamine phalloidin (R415, Invitrogen, USA) overnight at 4 ℃ in the dark, followed by incubation with Fluor488-conjugated secondary antibodies (Invitrogen, CA, Cat No. A21202) after three washes with PBS. Each coverslip was mounted with a medium containing the nuclear stain DAPI (Vectashield) (H 1200, Vector Laboratories, USA). The fluorescence images were obtained with a Laser Scanning Confocal Microscope (LSM 780, ZEISS, Germany) and visualised with the 100× oil immersion objective lenses (EC Plan-Neofluar 100×/1.3 oil).

### Luciferase reporter assay

Cells (2 × 10^5^ cells/well) were seeded into a 6-well plate and cultured overnight. The cells were then transfected with the luciferase reporter vector pGL4.37-ARE-Luc using Gene-Fect Transfection Reagent (TLC-001, TransLab, South Korea) according to the manufacturer’s instructions. The transfected cells, with or without brusatol treatment for 24 h, were harvested and lysed. Luciferase assays were performed on the lysate using the Dual-Luciferase Reporter Assay Systems (E1960, Promega, USA). The activity of the firefly luciferase was normalised to Renilla luciferase activity and was assessed using the Enspire Multi Plate Reader (PerkinElmer, USA).

### Statistical analysis

Results were indicated as the means ± standard deviations (SDs) from at least three independent experiments. Comparisons between groups were performed using a two-tailed Student’s t-test or ANOVA test. If p-value obtained by one-way ANOVA is < 0.05, the p-values between the groups are compared with post-test, Bonferroni, and Tukey HSD. p-values ≤ 0.05 were considered statistically significant.

## Supplementary Information

Supplementary Information.
