# Supplementary material for: Nrf2 regulates cell motility through RhoA–ROCK1 signalling in non-small-cell lung cancer cells
Source: Sci Rep. 2021 Jan 13;11:1247. doi: 10.1038/s41598-021-81021-0 (PMC7806835; doi:10.1038/s41598-021-81021-0)
Supplement: Supplementary file 1 — Supplementary Information. [file 41598_2021_81021_MOESM1_ESM.pdf]

Nrf2 regulates cell motility through RhoA-ROCK1 signalling in non–small-cell lung cancer cells

Eunsun Ko[1,2], Dasom Kim[1,2], Dong Wha Min[1], Seung-Hae Kwon[3] & Ji-Yun Lee[1,*]

Supplementary Figures

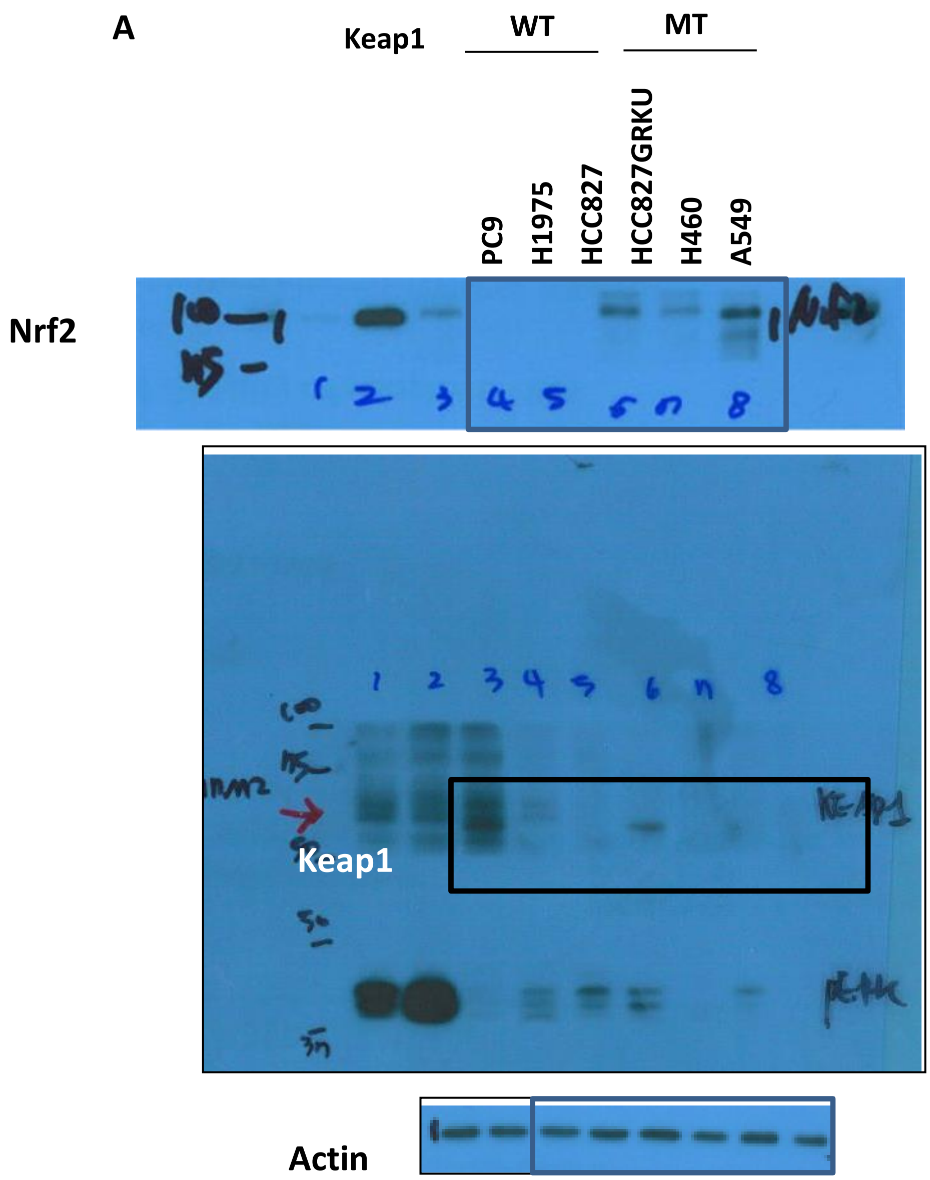

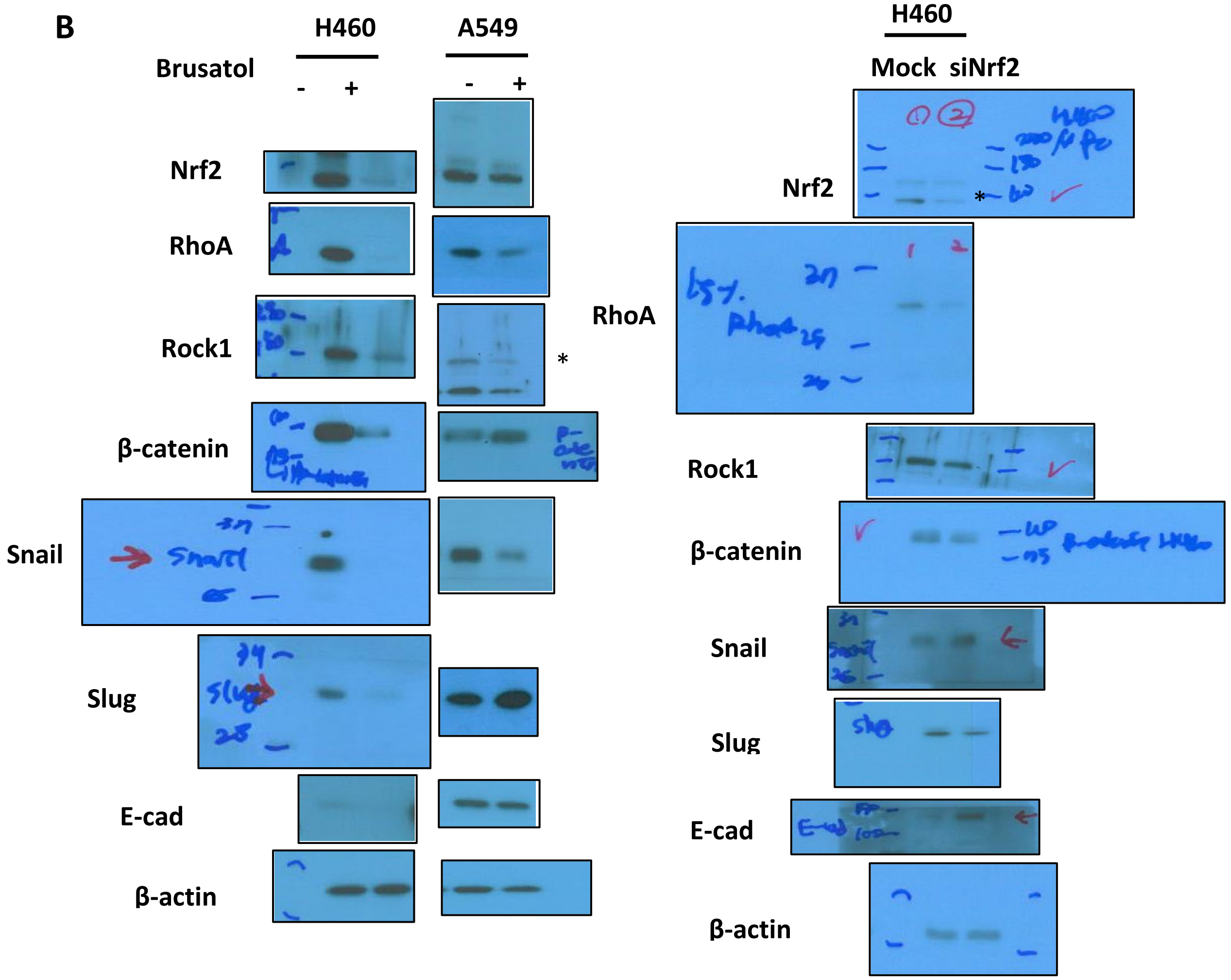

B
H460
A549
Brusatol
- +
- +
Nrf2
RhoA
Rock1
*
β-catenin
Snail
Slug
E-cad
β-actin
H460
Mock siNrf2
Nrf2
*
RhoA
Rock1
β-catenin
Snail
Slug
E-cad
β-actin

C

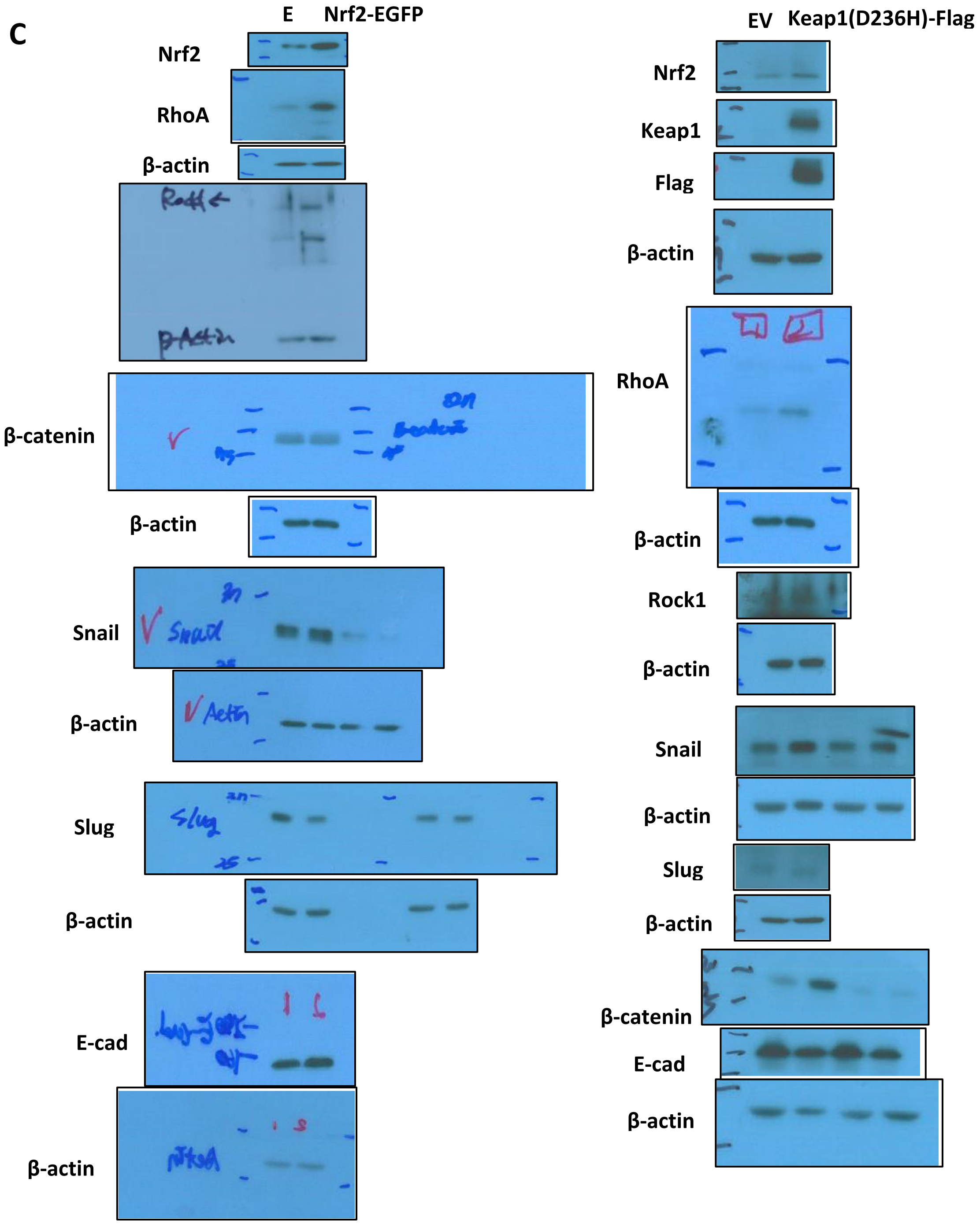
E
Nrf2-EGFP
Nrf2
RhoA
β-actin
β-catenin
β-actin
Snail
β-actin
Slug
β-actin
E-cad
β-actin
EV
Keap1(D236H)-Flag
Nrf2
Keap1
Flag
β-actin
RhoA
β-actin
Rock1
β-actin
Snail
β-actin
Slug
β-actin
β-catenin
E-cad
β-actin

D

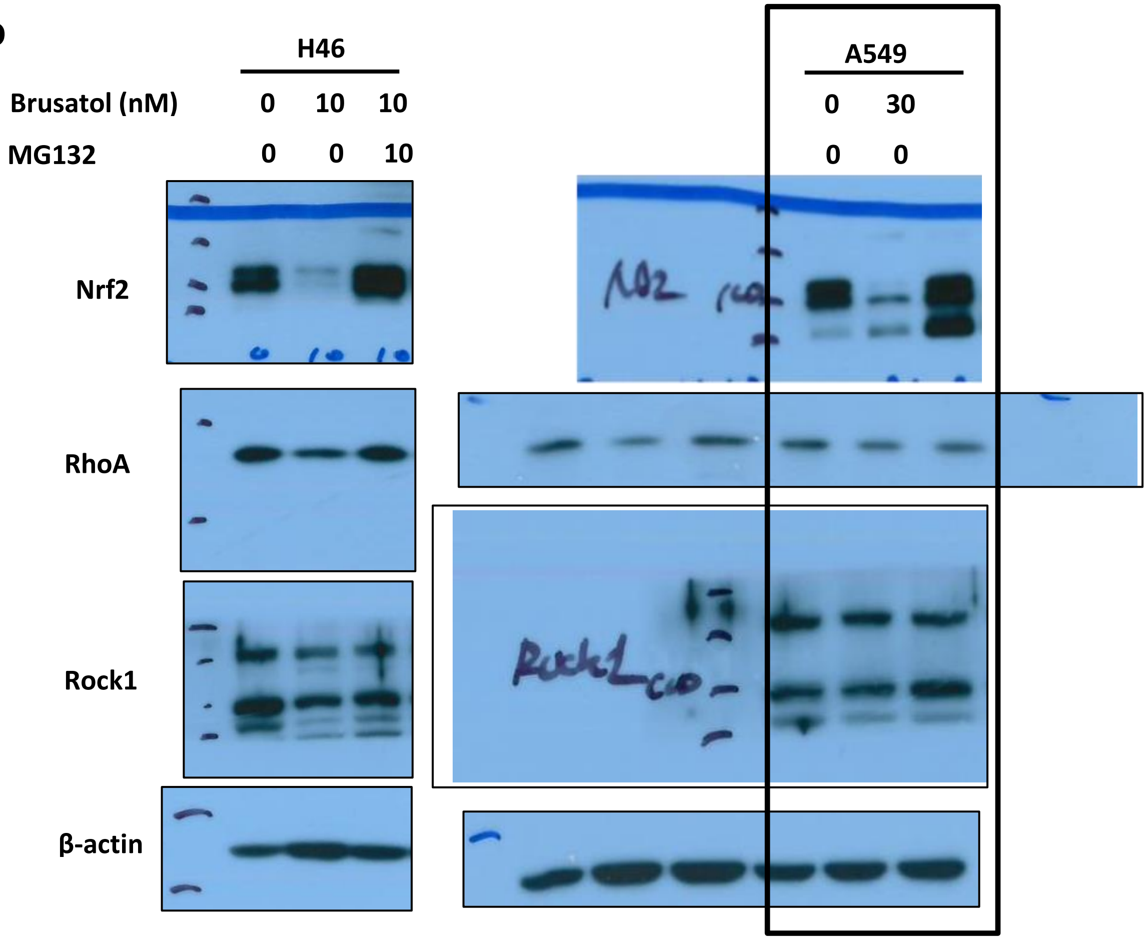
H46
A549
Brusatol (nM)
0 10 10
0 30
MG132
0 0 10
0 0
Nrf2
RhoA
Rock1
β-actin

E

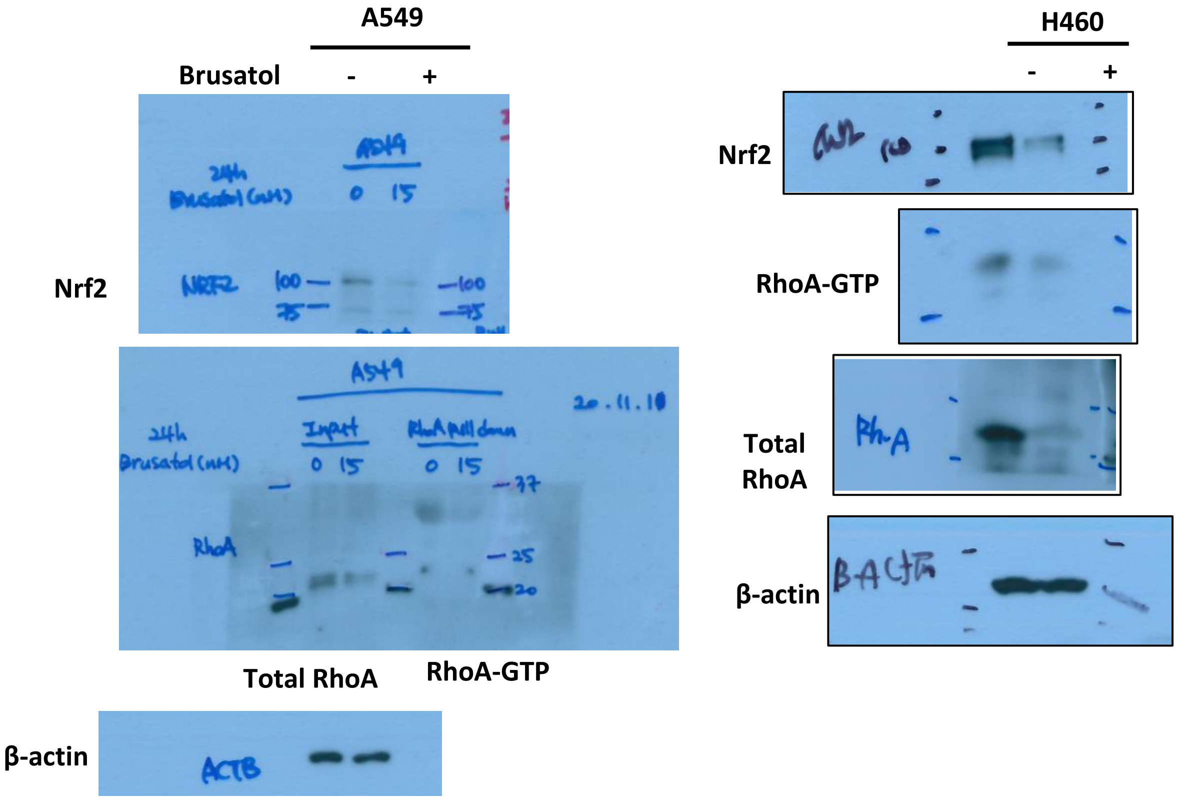
A549
Brusatol
-
+
Nrf2
Total RhoA
RhoA-GTP
β-actin
H460
-
+
Nrf2
RhoA-GTP
Total
RhoA
β-actin

F

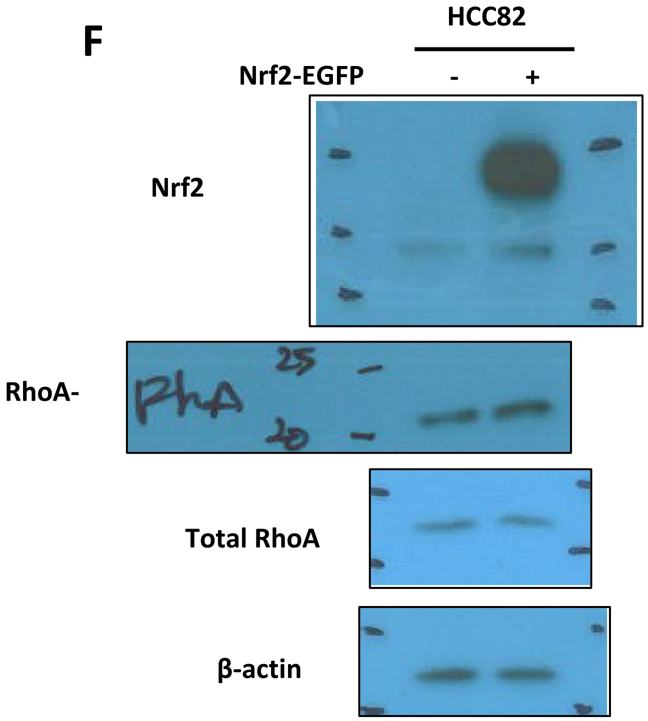
HCC82
Nrf2-EGFP
-
+
Nrf2
RhoA-
Total RhoA
β-actin

Supplementary Figure 1. Full-length blots of Fig.1a (A), Fig.3.a (B), Fig.3c (C), Fig. 4a (D), Fig.4.b (E), and Fig.4.c (F). Black box indicated image used in figures.
